# Multiport Circular Polarized RFID-Tag Antenna for UHF Sensor Applications

**DOI:** 10.3390/s17071576

**Published:** 2017-07-05

**Authors:** Jamal Zaid, Abdulhadi Abdulhadi, Arun Kesavan, Yassin Belaizi, Tayeb A. Denidni

**Affiliations:** 1 Institut National de la Recherche Scientifique Centre Centre Énergie Matériaux Télécommunications 800, De La Gauchetière Ouest Bureau 6900, Montréal, QC H5A 1K6, Canada; abdulhadi.abdulhadi@mail.mcgill.ca (A.A.); arun.kesavan@emt.inrs.ca (A.K.); denidni@emt.inrs.ca (T.A.D.); 2 IES-UMR CNRS 5214 Université de Montpellier, 34095 Montpellier Cedex 5, France; belaizi@ies.univ-montp2.fr

**Keywords:** radio frequency identification (RFID), ultra-high frequency, reading range, sensor, passive tag antenna, multiport, circular polarization

## Abstract

A circular polarized patch antenna for UHF RFID tag-based sensor applications is presented, with the circular polarization (CP) generated by a new antenna shape, an asymmetric stars shaped slotted microstrip patch antenna (CP-ASSSMP). Four stars etched on the patch allow the antenna’s size to be reduced by close to 20%. The proposed antenna is matched with two RFID chips via inductive-loop matching. The first chip is connected to a resistive sensor and acts as a sensor node, and the second is used as a reference node. The proposed antenna is used for two targets, serving as both reference and sensor simultaneously, thereby eliminating the need for a second antenna. Its reader can read the RFID chips at any orientation of the tag due to the CP. The measured reading range is about 25 m with mismatch polarization. The operating frequency band is 902–929 MHz for the two ports, which is covered by the US RFID band, and the axial-ratio bandwidth is about 7 MHz. In addition, the reader can also detect temperature, based on the minimum difference in the power required by the reference and sensor.

## 1. Introduction

UHF radio frequency identification (UHF-RFID) systems have become an attractive research topic in wireless communication networks due to the rapid expansion of RFID applications, including their use in asset tracking, security systems, remote wireless identification, purchasing, supply chain management, Internet of Things (IoT), and wireless sensor networks (WSNs) [1,2,3,4,5,6,7,8,9]. An RFID system consists of two parts, a reader and a tag. There are three kinds of RFID tags: passive, semi-passive, and active. Passive RFID tags have a long lifetime and are low-cost compared to active RFID tags. However, their reading range is shorter than that of active RFID tags. Basically, a passive RFID tag receives a signal from an RFID reader and then transmits a backscatter signal to the reader, allowing the reader to read the tag code. The UHF RFID frequency band is divided into sub-bands; each region has a specific frequency band such as the bands 908–914 MHz, 950–956 MHz, and 902–928 MHz utilized in South Korea, Japan, and North America, respectively.

Microstrip antennas have many attractive features, including their planar structure, low cost, easy fabrication, and light weight, which have made them extremely popular and has led to their use in wireless communication networks, especially in RFID applications. RFID tags usually use a linear polarization (LP) [10,11,12,13]; however, these tags have a random orientation because they are commonly hand-held. To overcome this issue, the reader should use circular polarization (CP) to broaden the ability of the reader to receive signals from these tags independently of their orientation. In addition, a mismatch of polarization could occur between readers and their tags, causing a loss of 3 dB from the signal, as well as reducing the reading range roughly 41% compared to a matched polarization [4]. Different techniques have been used to resolve this mismatch and to achieve CP operation for tags [1,2,3,4,5,6,7,8,9]. RFID-enabled multiple ports and sensor systems have become extremely attractive, offering a challenging topic for researchers due to their varied and useful applications [14,15,16,17,18,19,20,21,22].

To overcome the mismatch problems, a new antenna structure is proposed here, an asymmetric-star shaped slotted microstrip patch antenna (ASSSMP). This ASSSMP antenna can be etched on a patch to achieve CP operation, and it reduces the antenna size by roughly 20% compared to a conventional microstrip antenna. The proposed antenna is matched with two chips, one of which is used as a reference node and other connected to a sensor. The proposed antenna is matched with two ports, which eliminates the need for two antennas, as in [14,15,16,17]. While another work has also proposed using a single antenna that uses two ports [18], that one requires solar cells and a microcontroller, making its structure much more complicated than the one proposed here. Moreover, its patch size is larger and it is LP rather than CP. The proposed antenna has a reading range of about 25 m, achieved by using a linear-polarized reader (GAO). The proposed antenna has many desirable features, such as a simple structure, easy fabrication, low cost, compact size, is self-powered, and uses circular polarization, which means the reference node and sensor are in the same environmental conditions and receive the same power from the two reader chips at the same time and at any orientation. This aspect of similar conditions allows the use of a single antenna for the reference node and the resistive sensor, which improves the accuracy and sensitivity of the sensor system. The proposed antenna operates at the North America band (902–928 MHz), and it is matched with two tags at the center frequency of this band (915 MHz) by inductive loop matching. The sensor is operated based on the power difference between the reference node and the resistance node.

This paper is organized in six sections. Section 1 is a review of RFID systems and sensors. The principles of the CP-ASSSMP are explained in the Section 2, followed by a description of the proposed antenna design in Section 3. Section 4 presents the RFID-based sensor concept, and Section 5 presents the simulated and measured results. Finally, the conclusions are presented in Section 6.

## 2. CP-ASSSMP Design

**Figure 1:**
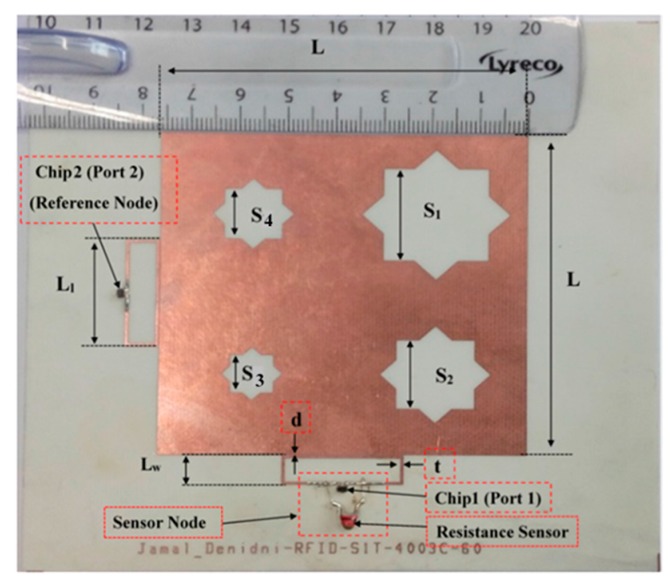
Photo of the fabricated antenna.

**Figure 2:**
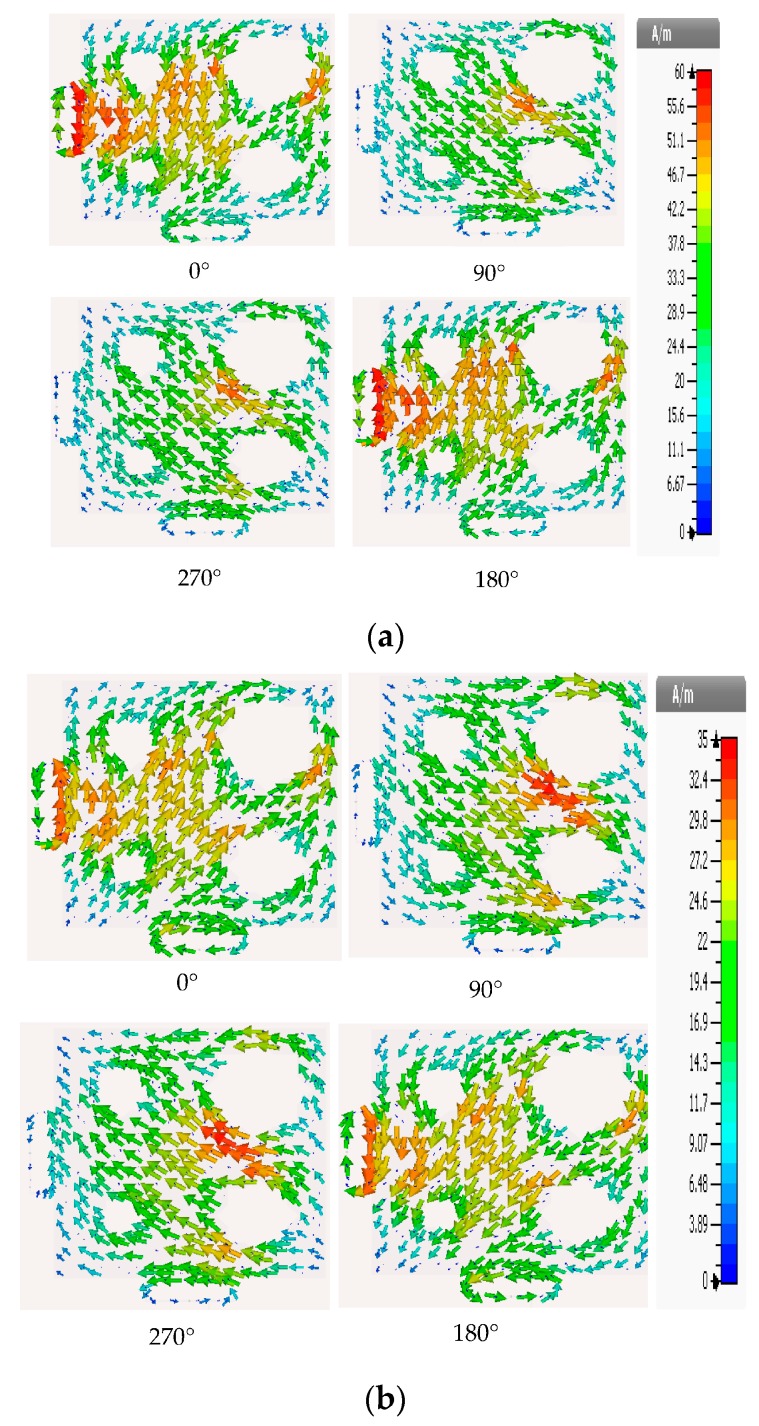
Simulated surface current distribution of the proposed antenna for (**a**) port one and (**b**) port two.

Since RFIDs are generally used in portable or handheld systems, CP is preferable, because CP can receive a signal at any orientation (orientation diversity), thereby increasing the antenna’s ability to receive a signal and reducing multipath effects. The CP asymmetric-stars shaped slotted microstrip patch antenna (ASSSMP) was therefore designed to provide CP. Figure 1 shows the proposed antenna. The antenna is printed on an RO4003 substrate (ɛr = 3.55, tan (δ) = 0.0027 and thickness = 1.52 mm) and four stars are etched on the patch, as shown in Figure 1. The etched stars reduce the patch size and generate CP. They change the distribution of the current on the patch; the current increases the current path length, thereby increasing the electric size of the antenna, as well as shifting the resonance frequency downwards. Moreover, by slightly changing the size of the etched stars, two modes are created perpendicular to each other, generating CP. Figure 2 shows how much the simulated distribution surface current is changed, with four phase angles of 0°, 90°, 180°, and 270°.

**Figure 3:**
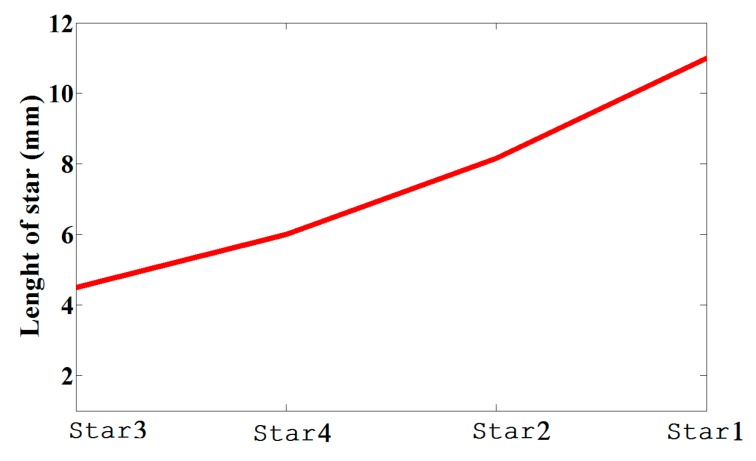
The relation between the stars’ sizes.

**Figure 4:**
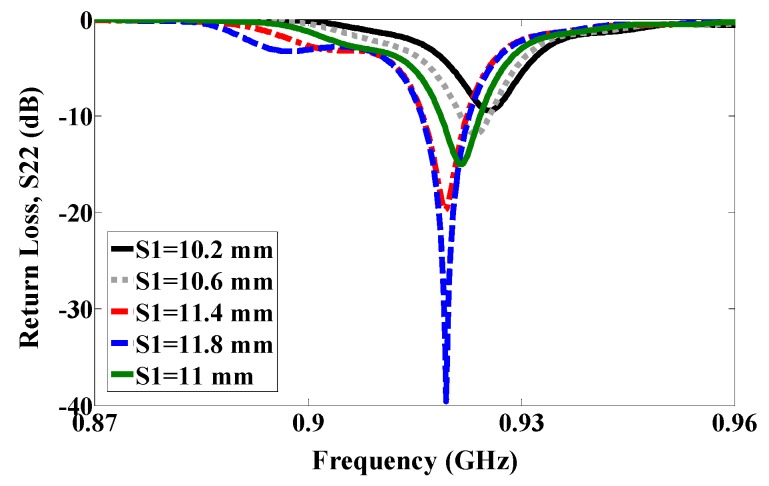
Simulated return loss.

**Figure 5:**
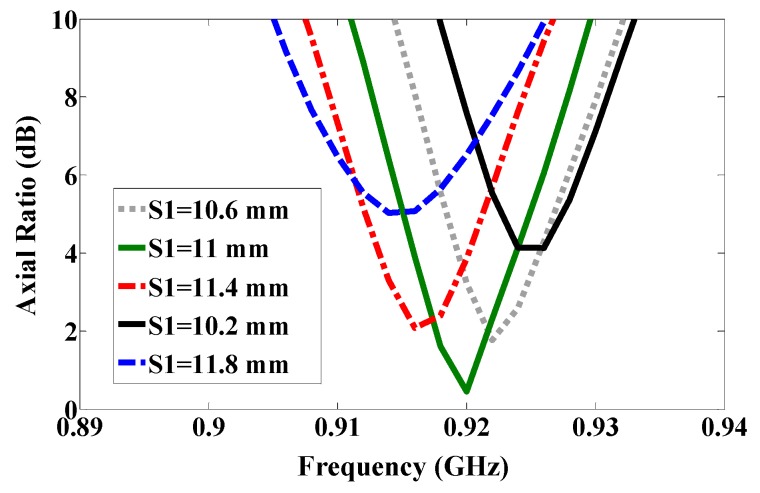
Axial ratio.

**Figure 6:**
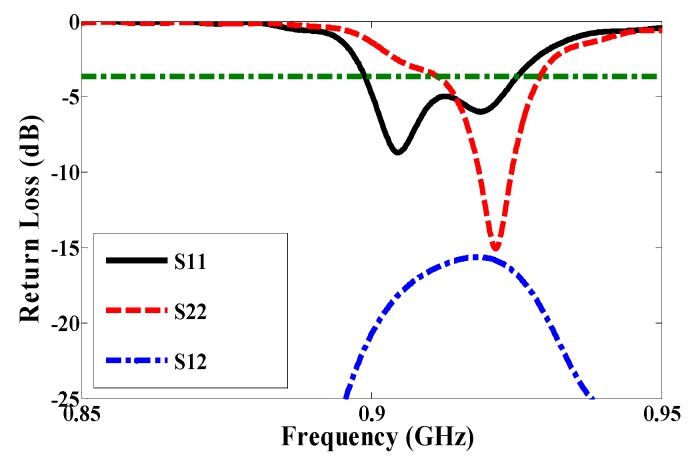
Simulated return loss of the proposed antenna.

By using symmetric slot stars, the operating frequency is shifted downwards without CP. However, Asymmetric slot stars can achieve both a reduction in the size of the microstrip antenna and the CP. The position of the stars in the middle of each quarter and their size are defined as an exponential function as shown in Figure 3. Figure 4 shows how increasing the size of one star, (S1), shifts the resonance frequency down to a lower frequency. In addition, the axial ratio based on the electric size of the antenna increases by increasing the slot size as shown in Figure 5. From our parametric study, the best values of S1 are between 10.6 mm and 11.4 mm. Port one generates left-hand circular polarization (LHCP) and port two generates right-hand circular polarization (RHCP), as shown in Figure 2a,b, respectively. The CP ASSSMP is matched with two RFID chips by the inductive loop-matching technique; the inductive loops are designed to create conjugate matching (Z Antenna = Z*chip) between the antenna and the RFID chips. The RFID chip has an input impedance of (24 − *j*190) at 915 MHz, which is matched with the antenna by conjugating the impedance (24 + *j*190), as illustrated in Figure 6. The return losses of the reference node and the sensor node at half power cover the North America RFID band (902–929 MHz); and the coupling between the two ports at the same bandwidth is roughly −16 dB.

## 3. RFID Antenna Design

A CP antenna is suitable for RFID applications as it has the ability to receive a signal from any orientation. Many techniques have been used to design CP antennas [1,2,3,4,5,6,7,8,9]. The dimensions of the proposed antenna are L = 76.2 mm, S1 = 11 mm, S2 = 8.168 mm, S3 = 4.5 mm and S4 = 6 mm, as shown in Figure 1. The etched stars are created using two squares with a shift angle of 45°. The CP ASSSMA is fed by two ports as shown in Figure 1. Each port is represented by an RFID chip. These chips have resistance and high capacitance, which are matched with the antenna by an inductive loop-matching circuit. The dimensions of the inductive loop are Ll = 25.15 mm, Lw = 6.8 mm, d = 0.2 mm, and t = 0.6 mm, as depicted in Figure 1.

## 4. Sensor Design

A number of RFID sensor designs have been proposed recently [14,15,16,17,18]. Some of these use one antenna for the reference node and another for the sensor node, as in [14,15,16,17]. Although one antenna is used as both sensor and reference node in [18]. However, this application is quite complicated because it requires solar cells and a microcontroller. The antenna proposed here uses a single antenna for both the reference and the sensor nodes, and makes it simple to avoid the effect of random orientation because of its CP. The reference node and the sensor node receive the same power from the reader, and in the same environment. The patch antenna is matched with the two ports by an inductive-loop matching circuit. By changing the width or length of the loop, the imaginary part of the loop can be changed to match the imaginary part of the chip. The temperature sensor is operated depending upon the different minimum power levels required to activate the sensor and reference nodes. When the resistive sensor is changed, the matching at the sensor node will also be changed, but not as much as at the reference node, thereby changing the minimum power required to activate the sensor node. The temperature value can then be determined based on the difference in power.

## 5. Results and Discussion

### 5.1. The Passive RFID Antenna Results

**Figure 7:**
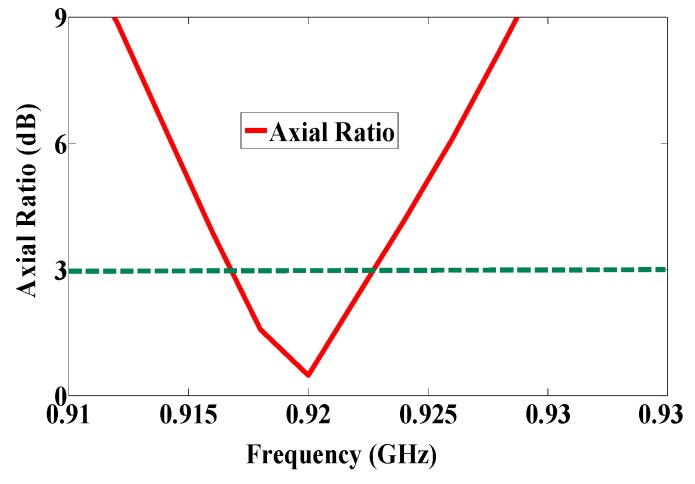
Axial ratio of the proposed antenna.

**Figure 8:**
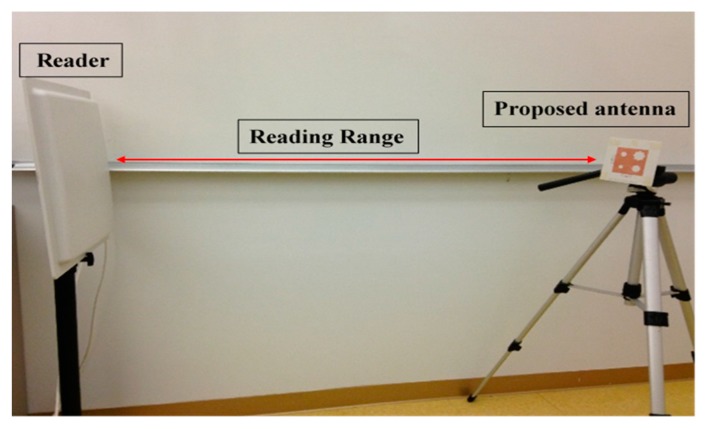
Reading range.

**Figure 9:**
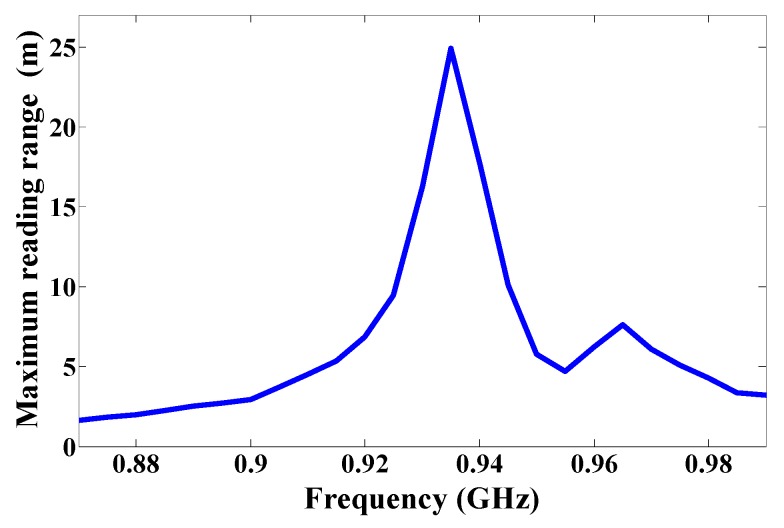
Reading range of the proposed antenna.

Using the etched slot technique, the proposed antenna is roughly 20% smaller than a conventional microstrip antenna. The asymmetric four stars on the patch increase the surface current path length more than a patch without slots. The electrical size of the antenna thus increases, while the resonance frequency shifts downward. Moreover, the four asymmetric etched stars generate CP, satisfied in a 90° phase difference, as shown in Figure 2. The return loss band at −3 dB for both ports covers in the US band 902–928 MHz, as shown in Figure 6, and the axial ratio bandwidth (ARBW) is about 7 MHz, as shown in Figure 7. The reader and the proposed antenna are adjusted as illustrated in Figure 8, and the distance between them is extended until the maximum reading range (25 m) is obtained, as illustrated in Figure 9. A linear polarized reader (GAO) is used in the all measurements. The proposed antenna has an acceptable gain and radiation efficiency of about 3.5 dB and 66%, respectively.

Table 1 shows how the proposed antenna compares with some earlier works in terms of bandwidth, dimensions, and reading range. As indicated, the reading range of the proposed antenna is longer than those found in the literature [1,3,4,5,7,8,9,18]. However, the proposed antenna is not the best in terms of bandwidth and size.

**Figure 10:**
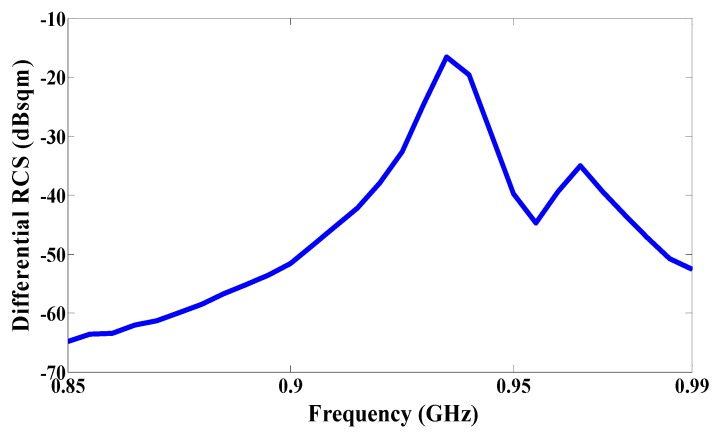
Measured differential radar cross-section as a function of frequency.

The radar cross-section can be calculated with Equation (1) in [10]. The received power (backscattered signal) from the tag is dependent on the radar cross section, and thus the received power is directly proportional to the radar cross section value. The radar cross-section results are plotted as a function of frequency in Figure 10, and the best value of the radar cross-section is −18 dBqm at a frequency 935 MHz.

(1) $$\Delta RCS=\frac{P_{received}{\left(4\pi \right)}^{3}d^{4}}{p_{t}G_{t}^{2}\lambda^{2}}$$

### 5.2. RFID Temperature Sensor Measurements

**Figure 11:**
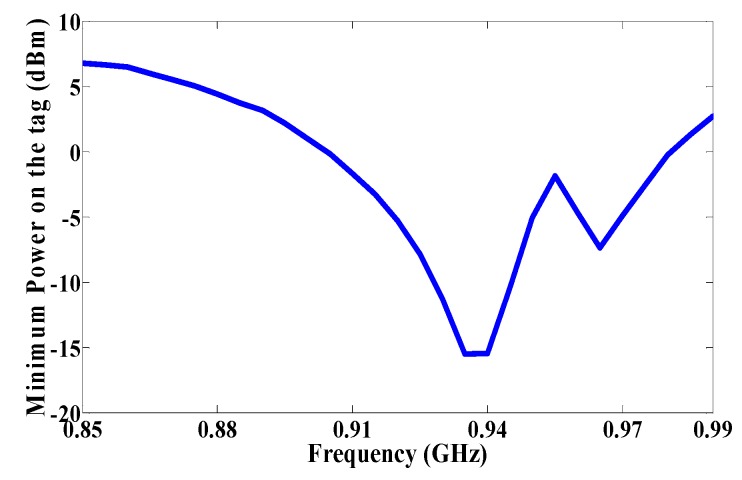
Measured minimum power required for activating the RFID chips.

**Figure 12:**
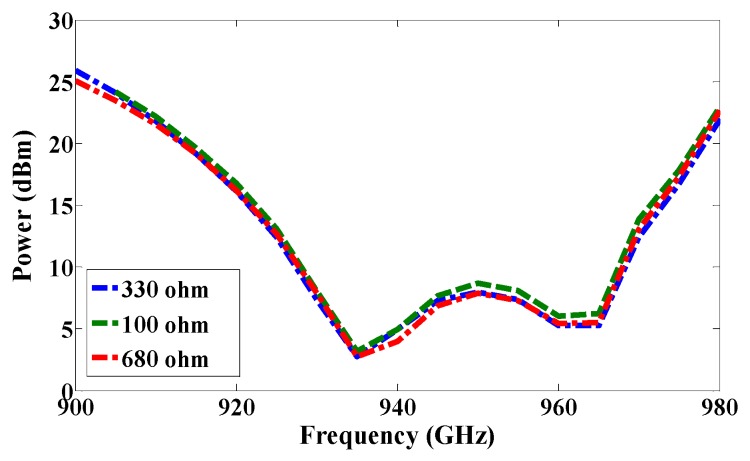
Measured minimum required power for activating the RFID chip at the reference node with changing resistance values.

**Figure 13:**
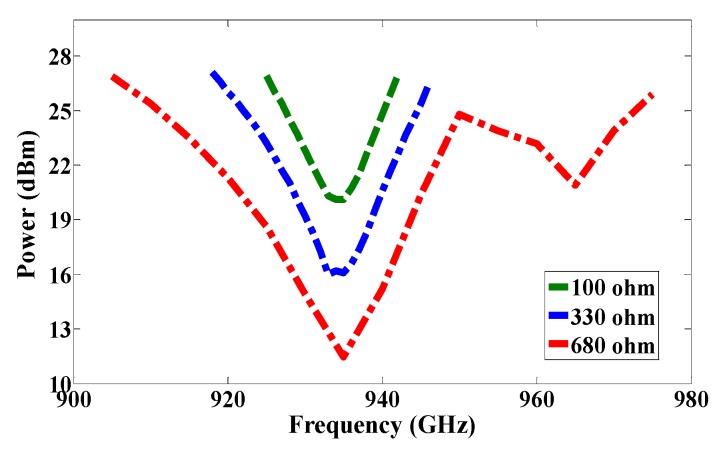
Measured minimum required power for activating RFID chip at the sensor node with changing resistance values.

**Figure 14:**
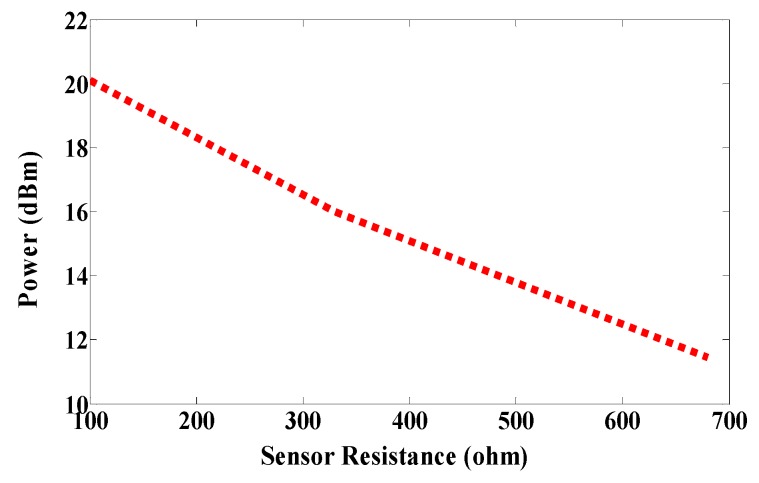
Measured power difference in the reference and sensor nodes.

The first RFID chip is connected in parallel with a resistive sensor; together they make up a sensor node, and the second RFID chip is used as a reference node. Both chips have the same power sensitivity, −20 dBm. As shown in Figure 5, the half-power bandwidth of the reference and the sensor nodes are 28 MHz and 32 MHz, respectively. The reference and sensor nodes are included in the same antenna, and so both nodes receive the same power from the reader and function under the same environmental conditions. By changing the temperature or humidity, the matching resistance value at the sensor node will change, and so the minimum power required for activating the RFID chip will change at the same node. However, the minimum required reference node power will not change because the matching does not change. The ratio of power for activating the reference and sensor nodes can be calculated by using Equation (2) [7]. The measured minimum required power to activate the tag is a function of frequency, as illustrated in Figure 11. Three different models of NTC thermistors were used in the measurements: NTCLE100E3331 (330 Ω), NTCLE100E3681 (680 Ω), and NTCLE100E3101JB0 (100 Ω), as the climate room was unavailable during the measurement procedure. The resistive sensors were soldered individually one by one. The minimum power required to activate the chip at the reference node and at the reference sensor were measured in each case, and the obtained results are presented in Figure 12. The minimum power at the reference nodes is almost the same for all cases. However, the minimum required power changes at the sensor node when the resistive sensor is modified because a mismatch occurs when the resistance changes, as shown in Figure 13. The minimum power required to activate the sensor node is 20.12 dBm, 16.18 dBm, and 11.45 dBm when the resistive sensor is 100 Ω, 330 Ω, and 680 Ω, respectively, as shown in Figure 13. The minimum power required for activating the tag is, thus inversely proportional to the resistance of the sensor. Considering the fabrication accuracy, the permittivity tolerance of ±0.05 and the parasitic effect of the resonance frequency shift to 935 MHz, the power difference between the reference and sensor nodes at frequency 935 MHz with different resistance values is calculated and plotted in Figure 14. Using the power curve ratio in Figure 14 to compare the minimum required power at the reference node makes it simple to determine the change in the temperature.

(2) $$P_{tmin}^{relative}=\frac{P_{t\;sensor}}{P_{t\;reference}}=\frac{\tau_{sensor\;node}}{\tau_{referencenode}}$$

## 6. Conclusions

A circular-polarized asymmetric stars-shaped slotted microstrip patch antenna (CP-ASSSMP) for UHF-RFID sensor applications has been designed, fabricated, and tested. A new antenna concept for generating CP and reducing the antenna size has been presented. The CP-ASSMP uses two RFID ports as reference and sensor nodes. The minimum required power to activate the reference and resistive nodes at three different resistance values have been measured and plotted. The sensor can detect temperature changes based on the differences in the power requirements. The reading range of the proposed antenna has been compared with several other antenna types. This new antenna matches the reference and sensor nodes to a single antenna, so they receive the same power under the same environment while eliminating the need for two antennas. Moreover, the reader can read both tags at the same time with a reading range of about 25 m without internal power requirements. Other key advantages of this antenna are its simple fabrication and low cost. Finally, the measured and simulated results show a good agreement.

## Figures and Tables

**Table 1:** Comparing the reading range of the proposed antenna with previous antenna designs.

References	1	3	5	7	8	9	18	Proposed Design
Operating frequency (MHz)	860–960	915–942	758–983	902–927	904–954	860–960	902–928	902–929
Dimensions mm^3^	189.6 × 127.9 × 21.6	58.6 × 58.6 × 0.4	250 × 250 × 33.8	100 × 100 × 1.6	70 × 70 × 1.6	54 × 54 × 5.2	130 × 130 × 1.52	130 × 130 × 1.52
Reading Range (m)	8	20.5	7	4.62	4.83	8.3	7.48	25
